# Molecular Detection of Antibiotic Resistance Genes in Shiga Toxin-Producing *E. coli* Isolated from Different Sources

**DOI:** 10.3390/antibiotics10040344

**Published:** 2021-03-24

**Authors:** Momna Rubab, Deog-Hwan Oh

**Affiliations:** 1 Department of Food Science and Biotechnology, College of Agriculture and Life Sciences, Kangwon National University, Chuncheon 200-701, Korea; rubab.momna@gmail.com; 2 School of Food and Agricultural Sciences, University of Management and Technology, Lahore 54770, Pakistan

**Keywords:** antibiotic resistance, phenotypes, genotypes, Shiga toxin-producing *Escherichia coli*, PCR

## Abstract

Shiga toxin-producing *Escherichia coli* (STEC) is an enteric pathogen associated with human gastroenteritis outbreaks. Extensive use of antibiotics in agriculture selects resistant bacteria that may enter the food chain and potentially causes foodborne illnesses in humans that are less likely to respond to treatment with conventional antibiotics. Due to the importance of antibiotic resistance, this study aimed to investigate the combination of phenotypic and genotypic antibiotic resistance in STEC isolates belonging to serogroups O26, O45, O103, O104, O111, O121, O145, and O157 using disc diffusion and polymerase chain reaction (PCR), respectively. All strains were phenotypically resistant to at least one antibiotic, with 100% resistance to erythromycin, followed by gentamicin (98%), streptomycin (82%), kanamycin (76%), and ampicillin (72%). The distribution of antibiotic resistance genes (ARGs) in the STEC strains was *ampC* (47%), *aadA1* (70%), *ere(A)* (88%), *bla_SHV_* (19%), *bla_CMY_* (27%), *aac(3)-I* (90%), and *tet(A)* (35%), respectively. The results suggest that most of the strains were multidrug-resistant (MDR) and the most often observed resistant pattern was of *aadA1*, *ere(A)*, and *aac(3)-I* genes. These findings indicate the significance of monitoring the prevalence of MDR in both animals and humans around the globe. Hence, with a better understanding of antibiotic genotypes and phenotypes among the diverse STEC strains obtained, this study could guide the administration of antimicrobial drugs in STEC infections when necessary.

## 1. Introduction

Shiga toxin-producing *Escherichia coli* (STEC) is a gram-negative and opportunistic bacterium and is a common inhabitant of the gastrointestinal tract of a wide variety of warm-blooded animals but may become pathogenic due to its easy dissemination in different ecosystems through the water, soil, food, and others [1,2]. In addition, it is also one of the world’s most studied bacteria and is arguably the best understood of all model microorganisms [3]. The gastrointestinal tract of ruminants, especially cattle and sheep, has been shown to act as a natural reservoir of STEC. STEC is characterized by a range of outcomes, from mild and self-limiting diarrhea to life-threatening clinical conditions such as bloody diarrhea, hemorrhagic colitis, and hemolytic uremic syndrome [4]. STEC is one of the global pathogens responsible for causing enteric infections in humans among the six known pathotypes of diarrheagenic *E. coli* (DEC) [5]. STEC’s ability to cause human injury is due to its ability to produce numerous virulence factors, especially Shiga toxin (*Stx*), which is one of the most potent toxins known to humans. Endothelial cells line the inner surface of blood vessels and are considered to be highly sensitive to STEC strains, which are cytotoxic to these cells [6]. More than 400 serotypes of STEC are recognized; however, serogroups O26, O45, O103, O111, O121, O145, and O157 are frequently associated with severe illness and outbreaks in humans, and colloquially termed the “top or big 7” [7].

Despite the tremendous risks posed by *E. coli*, the unfortunate emergence of resistance to known therapeutic antibiotics has raised challenges to the therapeutic treatment of *E. coli* infections [8]. Antibiotic treatment of STEC infections in humans is not recommended since there is evidence that treatment may worsen the disease by inducing toxin-related tissue damage and symptoms in patients [9]. However, toxin production depends on type and concentration of the antibiotic used [10]. In addition, it is widely accepted that extensive use of antibiotics in animal production systems is a major driver of multidrug resistance (MDR) in bacteria [10]. An alarming rise in the prevalence of MDR *E. coli* strains has been reported worldwide, and this is due to the spread of plasmids and other genetic elements. This has made antibiotic resistance a public health issue globally [8]. Furthermore, the inevitable evolution of resistance has undermined the great success achieved in the search for antibiotic agents and dashed man’s hope for recovery from infections and diseases since diseases and disease agents that were once thought to be controlled by antibiotics are now re-emerging in new leagues resistance to therapy [11]. The antibiotic resistance of foodborne bacteria should not necessarily be considered different from humans, food animals, or other niches isolates. Antibiotic resistance is one of the growing public health concerns among pathogenic and non-pathogenic bacteria [12]. *E. coli* is considered an indicator of antibiotic resistance and *E. coli* strains including STEC have been used for monitoring and surveillance of antibiotic resistance in animals, different environments, and humans. The development and dissemination of antibiotic-resistant *E. coli* strains has become a public health concern worldwide, as antibiotic-resistant STEC may be transmitted to humans through direct or indirect contact with the animals along the food chain through occupational exposure or manure runoff from cattle farms or through ingestion of a variety of contaminated food or water [13]. Integrons are important players in the dissemination of antibiotic resistance among Gram-negative bacteria due to their ability to capture, excise, and express genes, often included in mobile elements such as plasmids and transposons [14]. *E. coli* is a candidate vehicle for such transfers because of its diversity and also because it survives in the gastrointestinal tracts of both humans and animals as common flora [15]. In *E. coli*, the two main contributors to the bacterium intrinsic resistance are its outer membrane, which is impermeable to many molecules, and its expression of numerous efflux pumps, which effectively reduces the intracellular concentration of some antibiotics. Although the carriage of antibiotic resistance genes is not limited to commensal *E. coli* in the face of antibiotic selection, the ability to threaten human consumers is significantly enhanced if foodborne strains carry virulence genes that qualify them as potential human pathogens [15]. Moreover, antibiotic resistance causes prolonged illness, excess mortality with growing costs for patients and health care systems [16,17]. As discussed, *E. coli* have evolved different mechanisms to fight off the action of antibiotics, and in many cases a single strain can carry resistance genes to distinct classes of these agents, thus complicating treatment. The emergence of antibiotic resistance has been shown to be multifactorial, but all elements coincide in a major topic: antibiotic over abuse, both in human and veterinary medicine. After the era of plentiful antibiotics, we are alarmed by the increasing number of antibiotic-resistant strains. The genetic flexibility and adaptability of *E. coli* to constantly changing environments allow it to acquire a great number of antibiotic resistance mechanisms [18]. Despite increased warnings and numerous efforts to contain it, antibiotic resistance has been increasing [19].

In this paper, we determined the phenotypic and genotypic source of MDR in STEC isolates recovered from different sources. This inquiry seeks to deliver useful evidence on the prevalence and antibiotic resistance gene (ARG) profile of STEC isolates belonging to serogroups O26, O45, O103, O104, O111, O121, O145, and O157 from different sources. 

## 2. Materials and Methods

### 2.1. Bacterial Strains and Growth Conditions

In this study, a total of 51 strains were examined, kindly provided by the US Food Fermentation Laboratory Culture Collection (USDA ARS, Raleigh, N.C., USA) and the USDA ARS Eastern Regional Research Center (Wyndmoor, Pa., USA) (Table 1). All STEC strains were subculture in Luria-Bertani broth (LB; Difco, Becton, Dicknison, MD, USA) for 16–18 h at 37 °C.

### 2.2. Antibiotic Susceptibility Testing

Antibiotic susceptibility profiles of the isolated STEC strains were determined by disc diffusion method according to the standard procedure by American Clinical Laboratory Standards Institute (CLSI, 2015). The isolates were screened for susceptibility to a panel of thirteen different antibiotics belonging to nine classes, and *E. coli* ATCC 25922 was used as a control strain. The classes of antibiotics used in our assay were as follows: penicillin (ampicillin: A), carbapenems (imipenem: I, meropenem: M), aminoglycosides (streptomycin: S, kanamycin: K, gentamicin: GM), phenicols (chloramphenicol: C), tetracycline (tetracycline: T), glycolcyclines (tigecycline: TIG), lincosamides (clindamycin: CLI), macrolides (erythromycin: E), and fluoroquinolones (nalidixic acid: NA, ciprofloxacin: CIP). The 100 µL of inoculum for antibiotic susceptibility pattern testing was spread plated onto Muller-Hinton agar plates. The loaded antibiotics were placed on the inoculated plates under sterilized conditions using a safety bench. The plates were inverted 15 min after the discs and incubated at 37 °C for 24 h. The zone of inhibition diameters was measured in the millimeter and recorded. Each isolate was recognized as susceptible (S), intermediate (I), and resistant (R) to antibiotics according to the zone diameter interpretation standard recommended by the CLSI to establish the antibiogram profiles of the isolates.

### 2.3. Genomic DNA Extraction

The genomic DNA was extracted by using a commercial bacterial DNA extraction kit (AccuPrep^®^ DNA Extraction Kit (Bioneer, Daejeon, Korea) according to the kit manufacturer’s instructions. Briefly, 1 mL of STEC isolate (grown overnight in LB broth; Difco^TM^ Becton and Dickson and Company) was pelleted in Eppendorf tube by microcentrifuge at 10,000× *g* at max speed for 5 min. The supernatant was discarded followed by the addition of 200 µL of CL buffer and vortexed to completely resuspend the cell pellet. Then, 20 µL of proteinase K solution was added, samples were vortexed, and the cell solution was incubated at 56 °C for 15 min. After the lysis, 200 µL of BL buffer was added and vortexed and tubes were incubated at 70 °C for 10 min. The genomic DNA was concentrated by the addition of 200 °C for 15 min of absolute ethanol, pulse vortexed and centrifuged at 6000× *g* for 1 min. This was followed by the addition of 600 µL of BW buffer and 700 µL of TW buffer for washing and centrifuged at maximum speed for 1 min, respectively. The purified DNA was eluted in a fresh 1.5 mL microcentrifuge tube using 100 µL of elution buffer, kept at room temperature for 1 min, and centrifuged at full speed for 1 min. Finally, the DNA concentration was evaluated using a nanodrop spectrophotometer before being stored at −20 °C.

### 2.4. Detection of Antibiotic Resistance Genes

The phenotypic antibiotic-resistant STEC isolates were analyzed for the presence of relevant resistance genes using simplex PCR assay. The seven antibiotic resistance genes, including streptomycin (*aadA1*; adenylyl transferases), tetracycline (*tet*(A); efflux pump resistance), gentamicin (*aac(3)-I*; aminoglycoside acetyltransferases), beta-lactams (*bla*_SHV_; β-lactamase encoding penicillin resistance,* bla*_CMY_; β-lactamase encoding cephalosporin resistance), ampicillin (*ampC*), and erythromycin (*ere*(A); erythromycin esterase), were the selected target genes. The specific primer sequences, estimated size of the amplified products, and PCR conditions for targeted antibiotic resistance genes are depicted in Table 2 (Bioneer, Daejon, Korea). The amplification reactions were carried out using a C1000 Touch^TM^ thermocycler (Bio-Rad, Hercules, CA, USA). The amplified samples were analyzed by electrophoresis (Mupid-exU, Mupid, Tokyo, Japan) in 1.5% agarose gel, and the gel was visualized using a UV transilluminator (Gel Doc 2000; Bio-Rad, Hercules, CA, USA). A molecular weight marker with 100 bp increments (100 bp DNA ladder; ThermoFisher Scientific, Seoul, Korea) was used to determine the size of the PCR product.

## 3. Results

### 3.1. Antibiotic Resistance and Suceptibility Phenotype Characteristics 

**Figure 1:**
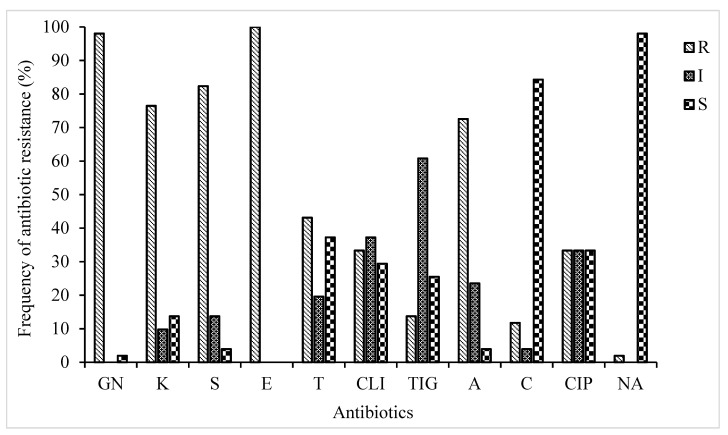
Antibiotic resistance profile of STEC isolates; GN: Gentamicin, K: Kanamycin, S: Streptomycin, E: Erythromycin, T: Tetracycline, CLI: Clindamycin, TIG: Tigecycline, A: Ampicillin, C: Chloramphenicol, CIP: Ciprofloxacin, NA: Nalidixic acid.

The current investigation revealed a wide presence of phenotypic antibiotic-resistant STEC strains isolated from diverse sources. A total of 51 STEC isolates belonging to eight serotypes were profiled for their probable phenotypic resistance to thirteen different antibiotics selected across nine antibiotic families (Figure 1). Antibiotic susceptibility testing demonstrated that all the 51 isolates were resistant to one or more antibiotics and the resistance rate was highest for erythromycin (100%), followed by gentamicin (98%), streptomycin (82%), kanamycin (76%), and ampicillin (72%). Varied resistance for other antibiotics were recorded as follows; tetracycline (43%), clindamycin (33%), ciprofloxacin (33%), and tigecycline (13%). Conversely, none of the STEC isolates were resistant to imipenem and meropenem and so therefore excluded from the subsequent analyses. In addition, STEC strains were highly susceptible towards nalidixic acid (98%) and chloramphenicol (84%). The current study revealed resistance to numbers of antibiotics ranging from two to eight with the percentage of 1.96, 3.92, 19.6 21.5, 21.5, 13.7, and 17.6%, respectively. According to phenotypic antibiotic resistance tests, multiple resistance patterns (composed of a combination of antibiotics) were determined (Table 3). The most prevalent phenotypic MDR patterns for the STEC strains were for 5 and 6 antibiotics (43%). There was no significant difference (*P* > 0.05) in the multiple antibiotic-resistant phenotypes. 

### 3.2. Antibiotic Resistance Genes

**Figure 2:**
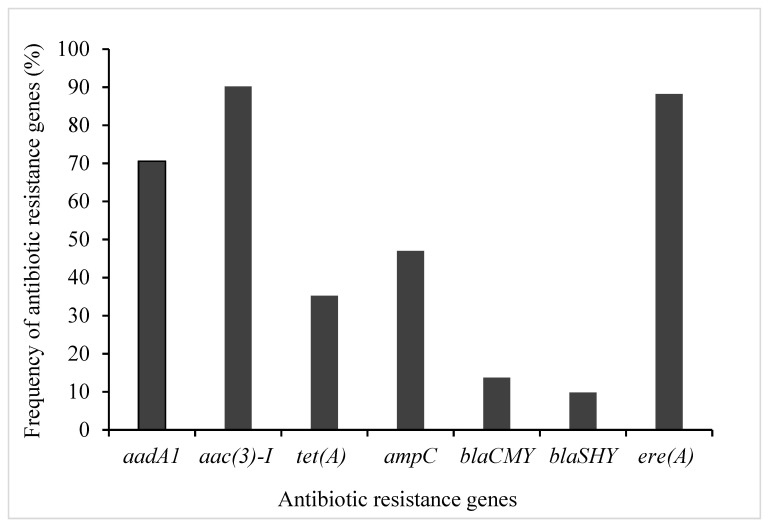
Antibiotic resistance gene profiles of STEC isolates.

PCR amplification was performed to detect antibiotic resistance genes using their appropriate primers (Table 2). The choice of the resistance genes assessed was based on their high frequency of occurrence in phenotypically resistant STEC isolates (Table 3 and Figure 1). Therefore, seven genes encoding for resistance to antibiotics in four families (aminoglycosides, β-Lactams, macrolides, and tetracycline) were screened for possible detection, prevalence, and distribution of resistance determinants among STEC isolates from diverse sources (Table 1). The distribution of identified antibiotic resistance genes presented in Figure 1. Antibiotic resistance profiling showed that most of the STEC strains exhibited the presence of all the tested antibiotics resistance genes (Figure 2). As shown in Figure 2, three ARGs belongs to extended-spectrum β-lactamases (ESBLs; *ampC*, and *bla_SHV_, bla_CMY_*) were tested to investigate the resistance of STEC isolates against β-lactam drugs (also originally called penicillinases). The results showed that STEC isolates show the highest presence of *ampC* genes with a frequency of 47%. The detection rates of *ere(A)* and *tet(A)* were 88, and 35%, respectively. Nearly 74.5% of those isolates were resistant to all of the tested antibiotic resistance genes.

## 4. Discussion

The emergence of antibiotic resistance among gram-negative bacteria is a worldwide challenge that affects human and animal health, further buttressing the need for intensified surveillance. Furthermore, STEC is considered one of the major challenges in both humans and animals at a worldwide scale and needs to be considered as areal public health concern [21]. In this study, the highest resistance was observed against erythromycin, gentamicin, streptomycin, kanamycin, and ampicillin, with 100%, 82%, 76%, and 72%, respectively. The lowest percentage of resistance belongs to tigecycline, chloramphenicol, and nalidixic acid, with 13%, 11%, and 2%, respectively. Overall, among 51 STEC isolates, 74.5% demonstrated resistance to more than 50% of the antibiotics tested. The antibiotic resistance patterns of the isolates observed in this study are in contrast with the results from previous studies [11]. With *E. coli* being the most widely studied bacteria, the resistance to at least two classes of antibiotic agents has been frequently documented [11]. Globally, the occurrence of MDR strains is a potential threat to the public health. 

Bacteria can be a reservoir of genes for antibiotic resistance and may play a role in the distribution of antibiotic resistance to other pathogenic and commensal bacteria [22]. In addition, *E. coli* is known as a very efficient reservoir for ARGs and can transfer those genes to other pathogenic bacteria [23]. The current study investigated the characterization of antibiotic resistance determinants in STEC isolates from diverse sources. Beta-lactams are well-known antibiotics and are characterized by low toxicity and used to treat a wide range of infections; however, the associated serious threat posed by their resistance cannot be overemphasized. In the current study, β-lactams *ampC*, *bla_SHV_,* and *bla_CMY_* genes were all observed in their respective resistant isolates and ampicillin resistance gene (*ampC*) was the highly prevalent gene. Our result for β-lactams-resistant genes are in agreement with previously reported studies [24,25]. Regarding tetracycline, tetracycline-resistant bacteria have been broadly distributed in the environment, and a number of resistance genes have been characterized to date. The present investigation screened the *tet(A),* and the findings show that 88% of the strains were positive for this gene; this result is in agreement with the previous studies, who reported the high frequency of *tet(A)* was the frequent tetracycline determinant in *E. coli* isolates [10,26,27]. In regard to genes encoding for resistance to aminoglycosides, (*aadA1* and *aac(3)-I*) were often detected in microbial communities. In this study, relatively high resistance was observed in the presence of streptomycin (*aadA1*) and gentamicin (*aac(3)-I*) among glycosides resistant isolates. We found that the phenotypic resistance pattern of STEC strains was supported by the genotypic resistance of STEC strain isolated from different samples followed by a high prevalence of genes of antibiotic resistance [28]; however, it was not fully supported. This could be attributed to the enzymes that reduce antibiotic efficacy by reducing the absorption and accumulation of antibiotics intracellularly, or increased antibiotic efflux [29]. Furthermore, this was probably because the expression of antibiotic resistance is probably resulted from other unspecified genes. The correlation between genotype (absence or presence of a resistance gene) and phenotype (sensitive or resistant) was high for aminoglycosides, erythromycin, and ampicillin. However, antibiotic-resistant phenotypes can emerge from several different genetic determinants, and each determinant may represent unique epidemiological characteristics [30]. For example, *bla* genes frequently coexist with other antibiotic resistance determinants and may also be correlated with mobile genetic elements, raising the possibility of MDR [31]. The plasmids containing *bla* are frequently aligned with transposons and integrons and often carry other resistance determinants concurrently, including *aad* or *aac* encoding aminoglycoside nucleotidyltransferase (or acetyltransferase) [32]. Numerous studies on virulence, antibiotic resistance, and molecular epidemiology of STEC have been published worldwide since the first study on foodborne STEC in humans was reported nearly 40 years ago. The co-existence of multiple individual resistance mechanisms in different combinations (e.g., efflux and ribosomal target protection mediating resistance to the same drug class) promotes the selection of MDR strains and at the same time confers a high degree of resistance. The majority of resistance genes encoding a wide variety of resistance mechanisms are carried by mobile genetic elements such as plasmids, transposons, and integrons [33], which favor the co-transfer of MDR phenotypes between commensals and pathogens, animals, and humans. Molecular epidemiological studies have shown that the presence of certain ARGs, including the genes that encode resistance against tetracycline (*tetA* and *tetB*), ampicillin (*ampC*), gentamicin (*aac(3)*–*IV*), and aminoglycosides (*aadA1*), is the key cause of antibiotic resistance in STEC [28,34]. Whether plasmids (conjugal or otherwise) contributed to the antibiotic resistance among our *E. coli* isolates is unknown. Several studies have recorded that the prevalence of antibiotic-resistant *E. coli* has increased since 1950 [35]. Apart from the previously mentioned mechanism of MDR, inactivation or enzymatic degradation of antibiotics and chemical transformation of antibiotic compounds by glycosylation, adenylation, acetylation, phosphorylation, and hydroxylation have also become steadily more apparent as causes of MDR [36]. Some of these mechanisms might be accountable for resistance observed among pathogens examined in this study. An alarming rise in the prevalence of MDR *E. coli* strains has been reported worldwide, and this is due to plasmid spread and other genetic elements. This has turned antibiotic resistance globally into a major public health issue [8]. *E. coli* is an important food safety and public health concern because of its pathogenicity and potential for MDR. The spread of antibiotic resistance has emerged as a significant public health concern, particularly in resource-constrained countries where lack of strict adherence to antibiotic policies has created a challenge for clinicians, primarily in prolonged hospitalized patients, to treat serious infections. Presence of STEC strains with phenotypic and/or genotypic resistance is especially relevant when it comes to establishing new antibiotic-based therapies for early-stage STEC infections in humans, which can help prevent serious sequelae [37]. 

In conclusion, the STEC isolates retrieved from diverse sources showed that phenotypic and genotypic expressions were quite different because genotypes do not always correspond with the phenotypic expression of individual isolates. Often, more than one gene was linked with a given phenotypic resistance. Among all the isolates, a slightly different distribution of resistance genes and MDR was observed. This is of great concern and demands caution in the indiscriminate and inappropriate use of antibiotic agents, and related compounds on animals and humans. However, all the 51 isolates were sensitive to imipenem and meropenem; therefore, these drugs could be drugs of choice in the treatment of STEC infections. These results have implications for the antibiotic selection for the empiric management of infections, continuous surveillance of antibiotic susceptibility patterns, and effective control of hospital infection. Thereby, the amount of data generated that may be used both to track the epidemiological situation of antibiotic resistance as well as for risk analysis will increase dramatically. Moreover, with a better understanding of antibiotic genotypes and phenotypes among the diverse STEC stains obtained, this study could guide the administration of antibiotic drugs in the clinical treatment of STEC infections when necessary. 

## Figures and Tables

**Table 1:** Shiga toxin-producing *Escherichia coli* (STEC) isolates analyzed in this study.

Serogroup	Serotype	No. of Isolates	Source
O26	O26:H11	7	H
O45	O45:NM, O45:H2, O45:H12	7	Co (calf), H, Go
O103	O103:H2, O103:H6, O103:H11, O103:H25	7	H
O104	O104:H2, O104:H4, O104:H7, O104:H21	7	H, Ca, Co
O111	O111:H-, O111:NM, O111:H8	7	H
O121	O121:NM, O121:H19	6	H
O145	O145:H-, O145:NM	7	H, Co
O157	O157:H7	3	H, Gb
Total		51	

H; human, Go; goat, Gb; ground beef, Co; cow, Ca; carcass.

**Table 2:** List of DNA oligonucleotides used in this study for PCR amplification.

Target Gene	Primers	Oligonucleotide Sequence (5′→3′)	Size (bp)	Reference
**Aminoglycosides resistance**				
*aac(3)*–*I *	*aac(3)*–*I*–F	CTTCAGGATGGCAAGTTGGT	286	[15]
	*aac(3)*–*I *–R	TCATCTCGTTCTCCGCTCAT		
*aadA1*	*aadA1*–F	TATCCAGCTAAGCGCGAACT	447	
	*aadA1*–R	ATTTGCCGACTACCTTGGTC		
**β-Lactams resistance**				
*ampC*	*ampC*–F	AATGGGTTTTCTACGGTCTG	191	[20]
	*ampC*–R	GGGCAGCAAATGTGGAGCAA		
*bla*_SHV_	*bla*_SHV_–F	TCGCCTGTGTATTATCTCCC	768	[15]
	*bla*_SHV_–R	CGCAGATAAATCACCACAATG		
*bla*_CMY_	*bla*_CMY_–F	TGGCCAGAACTGACAGGCAAA	462	
	*bla*_CMY_–R	TTTCTCCTGAACGTGGCTGGC		
**Macrolides resistance**				
*ere*(*A*)	*ere*(*A*)–F	GCCGGTGCTCATGAACTTGAG	419	[15]
	*ere*(*A*)–R	CGACTCTATTCGATCAGAGGC		
**Tetracycline resistance**				
*Tet(A)*	*Tet(A)*–F	GGTTCACTCGAACGACGTCA	577	[15]
	*Tet(A)*–R	CTGTCCGACAAGTTGCATGA		

**Table 3:** Pattern of distribution of antibiotic resistance in STEC isolates; GN: Gentamicin, K: Kanamycin, S: Streptomycin, E: Erythromycin, T: Tetracycline, CLI: Clindamycin, TIG: Tigecycline, A: Ampicillin, C: Chloramphenicol, CIP: Ciprofloxacin, NA: Nalidixic acid.

No. of Antibiotics	Multidrug Resistance Profile	No. of Bacterial Strain	Total. No (%)
2	A, E	1	1 (1.96)
3	A, E, GN	1	2 (3.92)
	GN, CIP, E	1	
4	GN, A, E, K	1	10 (19.6)
	GN, E, S, CLI,	1	
	GN, A, E, S	3	
	GN, E, CLI, CIP	1	
	GN, A, E, CIP	2	
	GN, E, K, S	2	
5	GN, K, E, S, T	1	11 (21.5)
	GN, K, E, S, CIP	2	
	GN, K, E, S, A	5	
	GN, K, E, S, C	1	
	GN, KN, E, CIP, A	1	
	GN, S, E, CLI, A	1	
6	GN, K, E, A, S, T	5	11 (21.5)
	GN, K, E, A, S, CLI	1	
	GN, K, E, A, S, TIG	2	
	GN, K, E, A, CLI, CIP,	1	
	GN, K, E, S, CLI, T	1	
	GN, K, E, S, TIG, T	1	
7	GN, A, E, T, K, S, CHL	1	7 (13.7)
	GN, A, E, T, K, S, CLI	1	
	GN, A, E, T, S, CLI, CIP	1	
	GN, A, E, T, K, S, CIP	1	
	GN, E, K, S, CLI, CIP, TIG	1	
	GN, E, K, T, S, CLI, CIP,	1	
	GN, A, E, K, S, CLI, CIP	1	
8	GN, A, E, K, T, S, C, TIG	1	9 (17.6)
	GN, CIP, A, T, E, K, S, CIP	1	
	GN, K, A, E, T, S, CLI, CIP	2	
	GN, K, A, E, T, S, CLI, TIG	1	
	GN, K, A, E, T, S, CLI, C	1	
	GN, K, E, T, S, CLI, CIP, C	1	
	GN, K, A, E, T, S NA, TIG	1	
	GN, K, A, E, T, S, C, TIG	1	

## Data Availability

Data are contained within article.
